# Expression of E-Cadherin, Leukemia Inhibitory Factor and Progesterone Receptor in Mouse Blastocysts after Ovarian Stimulation

**Published:** 2012-12-12

**Authors:** Bahar Movaghar, Saeedeh Askarian

**Affiliations:** 1. Department of Embryology, Reproductive Biomedicine Research Center, Royan Institute for Reproductive Biomedicine, ACECR, Tehran, Iran; 2. Department of Modern Sciences and Technologies, School of Medicine, Mashhad University of Medical Sciences, Mashhad, Iran

**Keywords:** Blastocyst, Ovarian Stimulation, Progesterone Receptor, Leukemia Inhibitory Factor, E-Cadherin

## Abstract

**Objective:**

The appropriate interaction between a blastocyst and the endometrium is essential for successful implantation. Numerous factors, including hormone receptors (progesterone receptor), cytokines [leukemia inhibitory factors (LIF)], and adherence molecules such as *E-cadherin* are involved in the cross-talk that occurs between the embryo and endometrium. Studies show that a lack of these genes impact endometrial receptivity. In this study, we compare the expression levels of *E-cadherin*, LIF, and progesterone receptor (*PgR*) genes in blastocysts that have been obtained from superovulated mice to those obtained from natural cycles.

**Materials and Methods:**

In this experimental study, for the experimental group, a total of 17 virgin female NMRI mice (6- 8 weeks old) were injected with 7.5 IU pregnant mare serum gonadotropin (PMSG). Their blastocysts (approximately n= 120) were flushed out after 3.5 days, following administration of human chorionic gonadotropin (hCG). The control group consisted of blastocysts from 62 female mice that were mated with male mice. The natural cycle blastocysts were flushed out from the female mice uteri 3.5 days after mating. The expression levels of *E-cadherin*, *LIF*, t *PgR* genes were examined by quantitative real-time reverse-transcriptase polymerase chain reaction (RT-PCR). Data were analyzed by the student’s t-test (one sample t-test).

**Results:**

Expression levels of all studied genes were significantly lower in the hormone-treated group compared to the natural cycle blastocysts (p<0.05).

**Conclusion:**

Although ovarian stimulation is utilized to obtain more oocytes in ART cycles, it seems that this could disadvantageous to implantation because of the decrease in expression levels of certain genes. Because of the important roles of E-cadherin, LIF, and progesterone receptor in the implantation process, we have shown lower expression levels of these genes in mouse blastocysts obtained from ovarian-stimulated mice than those derived from the natural cycle. The results observed in this study have shown the possibility of an unfavorable effect on implantation and pregnancy rate.

## Introduction

Appropriate interaction between a blastocyst and the endometrium is essential for successful implantation. Numerous factors are involved in the cross-talk between an embryo and endometrium, including hormones, growth factors, cytokines, and signaling pathways. While ovarian stimulation is used to obtain many oocytes, it seems that ovulation induction has some disadvantages for embryo growth and uterine receptivity, resulting in a decreased implantation rate (1, 2).

It has been presumed that the failure of embryo implantation and retarded fetal growth after superovulation is due to endometrial dysfunction, as the endometrial receptivity is sensitive to changes in the serum levels of sex steroids. The effects of exogenous gonadotropin on embryo quality remain controversial (3).

Ovarian stimulation also results in a delay in embryo development and an increase in post-implantation mortality in mice (4). It is also associated with a reduction in fetal growth and a shift in the window of receptivity in *in vitro* fertilization ( IVF) cycles (5). It has been shown that different ovarian stimulation approaches, can increase the chromosomal aneuploidies and influence on oocyte and embryo quality (6). Little is known about the genetic basis of the negative impact of gonadotropin stimulation on embryo development.

Steroid hormones play an important role in the development and implantation of pre-implantation embryos. However, it is controversial as to whether these hormones act directly on the embryos or not (7). In a comparison study by Microarray method, the expression level of 92 genes in the blastocysts of control and superovulated mice have been assessed. The results have shown that 76 genes were down-regulated in experimental group although these results need verification (8).

A gene expression analysis was performed on blastocysts which failed to implant in a uterus model *in vitro*. The expressions of some genes (*GADPH*) were lower than implanted blastocysts (8, 9). We can assume that gene patterns may directly correlate with successful implantation and pregnancy rate.

Pan and his colleagues have shown a decrease in expression of the estrogen receptor, progesterone receptor, and leukemia inhibitory factor (LIF) during different ovarian stimulation protocols in the endometrium, and that they may have an adverse effect on the endometrial receptivity in mice (10).

*LIF* is a cytokine that is presumed to be essential for the successful implantation of porcine, sheep, and primate trophoblasts (11). Blastocysts remain 'dormant' in *LIF (–/– )* mice (knocked out mice in *LIF*) and do not implant. It seems that *LIF* plays an important role during the invasion and implantation of mammalian embryos, and its mRNA expression might be directly or indirectly dependant on the interaction of progesterone with its receptor (12).

Initial adhesion is mediated by molecules that contribute specific carbohydrate ligand binding, including cadherins (13). Members of the cadherin superfamily mediate cell-cell interaction by calcium-dependent homotypic or heterotypic binding (14). E-cadherin is located at the lateral epithelial plasma membrane and is likely to be critical for the maintenance of adherent junctions (15). As E-cadherin is found on luminal epithelium (LE) and also on the trophoectoderm, it has been suggested that it may be involved in the initial attachment of the embryo (16). Hormones like progesterone are associated with tight junction proteins, for example it is has been proven that progesterone elicits transient decreases in tight and adherens junctions in the endometrial LE between days 10-12 in ewes (17).

It would appear that a considerable portion of implantation failure is attributed to the blastocyst. It is useful to characterize the effect of treatments such as ovarian stimulation on molecular pathways involved in the blastocyst-uterine cross-talk by taking into consideration implantation failure and early pregnancy loss. The objective of this study is to compare the expression levels of *E-cadherin*, *LIF*, and progesterone receptor (*PgR*) genes in blastocysts from superovulated mice to those from a natural cycle.

## Materials and Methods

### Embryo collection

In this experimental study, 17 virgin female NMRI mice (6-8 weeks old) were superovulated via intraperitoneal injection with 7.5 IU pregnant mare serum gonadotropin (PMSG) and 7.5 IU of human chorionic gonadotropin (hCG) at 48- hour intervals. The female mice then mated with male mice and were inspected for a vaginal plug the next morning. About 120 blastocysts were obtained from plug-positive mice, 3.5 days later and were considered to be the experimental group. In the control group, 62 female mice mated with males and 120 natural cycle blastocysts were flushed out from their uteri 3.5 days after mating.

All animal experiments were performed out according to the guidelines of the Iranian Council for Use and Care of Experimental Animals (affiliated to the Royan Institute).

### RNA extraction and reverse transcription

Total RNA was extracted from blastocysts (about 35 blastocysts per group) using an RNA extraction kit (Qiagene, Germany) in accordance with the manufacturer’s instructions. Total RNA was quantifi ed by spectrophotometry (Biowave II, WPA, UK). After treatment with RNase-free DNase (Qiagene, Germany), 1 µg of each sample was reverse transcribed using random hexamer primers (Fermentas, USA) and M-MLuV reverse transcriptase (Fermentas, USA), in a fi nal volume of 20 µL. Subsequently, cDNA samples were used as the template for polymerase chain reaction (PCR).

### Real-time reverse transcriptase polymerase chain reaction (RT-PCR)

RT-PCR was performed in triplicate on blastocyst samples to quantify and compare the mRNA expression pattern of E-cadherin, *LIF*, and *PgR* genes. The housekeeping gene *GADPH* was used as an internal control. Real-time RT-PCR (Applied Biosystems 7500 Fast Sequence Detection System; Applied Biosystems, Foster City, CA) was performed using SYBR Green according to the manufacturer’s instructions. After 10 minutes incubation at 95℃, amplification was performed for 40 cycles at 95℃ for 15 seconds and 60℃ for 1 minute. Next a dissociation stage was performed for 15 seconds at 95℃, 1 minute at 60℃, 15 seconds at 95℃, and 15 seconds at 60℃. Standard curves were generated to test assay effi ciency, sensitivity, and working range. Each reaction mixture contained SYBR Green mix (TaKaRa BIO, Shiga, Japan), forward and reverse primers and 1 µl of template (complementary DNA or cDNA) in a total volume of 20 µl. A "no template control" that contained water was included in each reaction. Expression changes were calculated using the ddCt method and the following primers were synthesized (Gene Blue, Canada), as shown in table 1.

### Statistical analysis

Data analyses were carried out by student’s t test (one sample t test) and Kolmogorov-Smirnov test using the SPSS software (version 11.5; Chicago, IL, USA, http://www.spss.com).

**Table1:** Sequence of primers used in the quantitative analysis of mRNA by real-time RT–PCR


Gene	Primer (forward 5'-3')	Primer (reverse 5'-3')

E-cadherin	GCTGGACCGAGAGAGTTAC	GGCACTTGACCCTGATACG
LIF	TGCTCTCTTCATTTCCTATTACAC	AACTTGGTCTTCTCTGTCC
PgR	CTAATCCTAAATGAGCAGAG	AATTGTGTTAAGAAGTAGTAAGAC
GAPDH	GACTTCAACAGCAACTCCCAC	TCCACCACCCTGTTG


## Results

The number of embryos obtained from each mouse in experimental group had a wide range in comparison with control group. The variable number of blastocysts is because of different responses of mice to hormonal treatment. For this reason mice with blastocyst number between 5 and 25 were mentioned in our study.

About 80 percent of obtained embryos in experimental group was late blastocyst but in control group almost 55 percent were late and others were early blastocyst (Data was not shown). This synchronization can be a result of ovarian stimulation.

**Fig 1:**
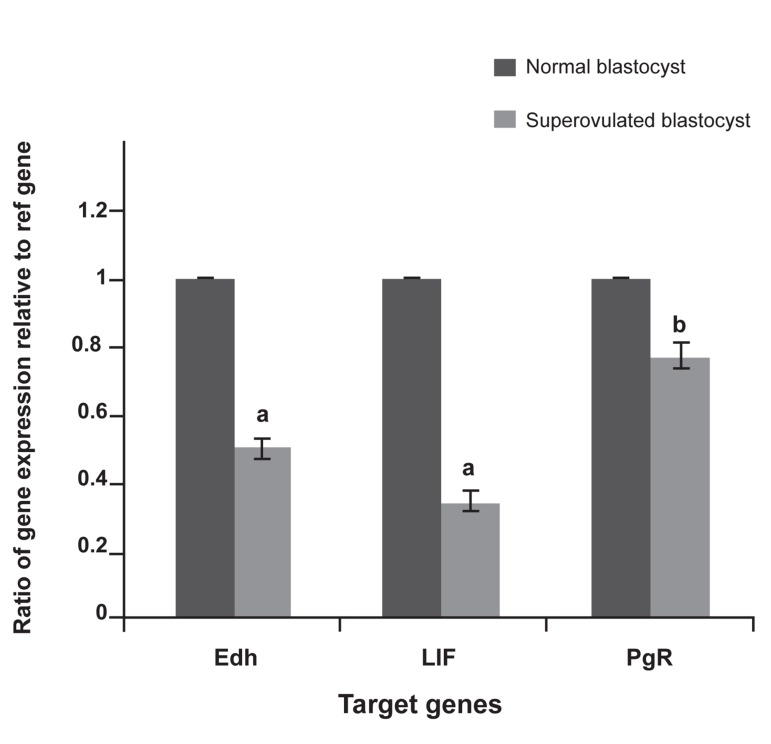
Real-time RT-PCR quantification results. Mean ± SD relative expression of E-cadherin (Edh), leukemia inhibitory factor (LIF), and progesterone receptor (PgR) mRNA calculated as percentage of expression of housekeeping gene (GAPDH) mRNA. (a: p<0.001, b: p<0.05).

 Expressions of *E-cadherin*, *LIF*, and *PgR* genes in mice blastocyst samples were evaluated with real-time RT-PCR. There was a significant decrease in expression of *E-cadherin* and *LIF* (p<0.001), and PgR (p<0.05) in blastocysts of stimulated mice compared with control blastocysts. The largest decrease in expression levels was seen in *LIF* and E-cadherin compared to the control group (Fig 1). Blastocysts of ovarian stimulation and normal groups showed no difference in their appearances.

## Discussion

Ovulation induction triggers the release of oocytes from already developed follicles. In ART techniques, it is beneficial for development, final maturation of the oocytes and proper timing of egg retrieval (18).

It has been reported that excessive ovarian stimulation is associated with a significant reduction in pregnancy rate and results in a shift of the receptivity window in IVF cycles. Ovarian hyperstimulation causes low endometrial receptivity and possibly weak embryo quality (3).

During early mouse embryogenesis the mRNAs of LIF, LIFR, and gp130 are undetectable in one- or two-cell embryos, but are present in the blastocyst stage (19). This pattern of expression is strongly suggestive of a paracrine action of LIF between the embryo and the uterus during the implantation window. Endometrial aspirations from infertile women contain less LIF than those obtained from fertile patients (20).

Some studies show that ovarian stimulation decreases LIF expression in the human endometrium (21). For example, Blitek’s study on the effect of gonadotropins on the endometrium during early pregnancy in pigs has shown that PMSG/hCG resulted in decreased expression of endometrial HoxA10, TGF-beta, LIF, and PGHS-2 on day 12 of pregnancy (22).

The E-cadherin-null mice show defective pre-implantation embryo development and failure to implant (23). Satterfield and colleagues have demonstrated decreasing of E-cadherin protein in the LE of ewes treated with P4 and RU486 (mifepristone, a modulator of progestin and glucocorticoid action) until day 12 after the onset of estrus (17).

An investigation of endometrial steroid receptors in 22 infertile women from an IVF fertilization treatment study showed satisfactory response to ovulation induction, but there was a significant reduction in the nuclear receptor level in both progesterone and estrogen receptors (24).

We know much about the roles of steroid hormones on uterus but there are few knowledge about its effect on blastocysts. However, some studies have shown that the administration of exogenous gonadotropin does not affect cleavage capacity or quality assessment of the resulting embryos (25), while other studies are not agree with this object (6). In Ertzeid et al. study, ovarian stimulation impaired embryo development to the blastocyst, reduced implantation rate, increased post-implantation fetal mortality, and decreased the mean weight of live fetuses (4).

There are not many studies about the effect of hormones on gene expression in blastocyst and its relationship with implantation rate. Ghaemi et al. have evaluated the effects of progesterone and ovarian stimulation on development and implantation rate of mouse embryos. They demonstrated that cultured embryos in the presence of different concentrations of progesterone did not improve the negative effects of superovulation on the implantation rate (26).

In our study, *LIF* down-regulated in blastocysts of ovarian-stimulated mice. Our study agreed with the results from Hambartsoumian’s study, which stated that progesterone significantly down-regulated the *LIF* gene and endometrial LIF secretion (27).

We also observed a decrease in PgR expression in experimental group blastocysts. Pan et al. have shown that different ovarian stimulation protocols in mice decreased the expression of estrogen and progesterone receptors on the endometrium (10). However, in contrast to our study, Develioglu et al.’s study showed that after hCG administration, controlled ovarian hyperstimulation caused the early expression of endometrial estrogen and progesterone receptors (28).

The E-cadherin–catenin adhesion complex is crucial for the polarization and function of epithelial cells and for the integrity of epithelial cell layers (23). Our observation regarding the effects of ovarian stimulation on decreasing E-cadherin gene expression support the results of a study by Revel et al., which has shown that E-cadherin expression was disrupted by repeated IVF failures. They concluded that endometrial-disrupted E-cadherin regulation might be the basis for repeated IVF failure in patients (29).

In the present study, no morphological assessment was carried out on blastocysts of the control and experimental groups. Zhang and coworkers have shown that ovarian stimulation impaired the quality of mice blastocysts and changed its morphology (8).

## Conclusion

This study proposes that ovarian stimulation can result in down-regulation of some critical genes in blastocyst implantation. This may be the reason for low implantation rates in ART cycles. The complete molecular dialogue and the order of events leading to the attachment of a competent blastocyst to a receptive uterine LE, in particular the role of the blastocyst, is still not fully understood.
